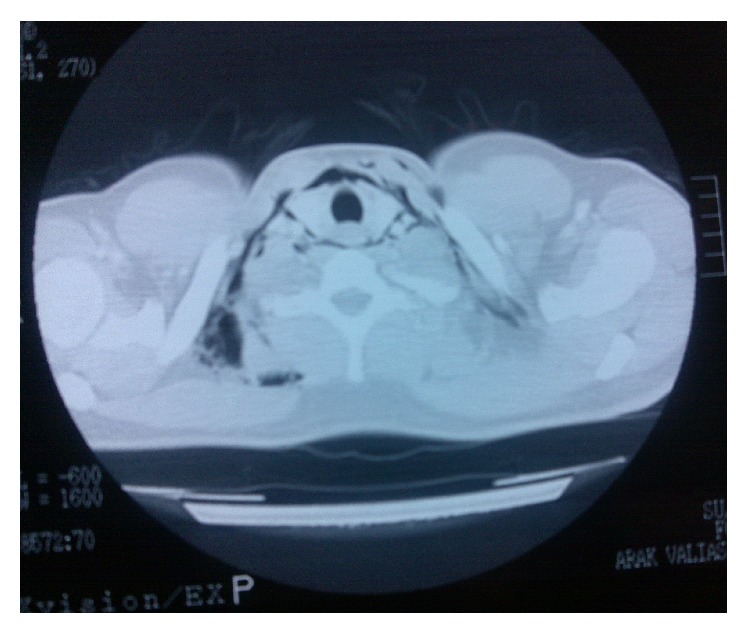# Pneumomediastinum following Crystal Use: A Report of Two Cases

**DOI:** 10.1155/2016/9730484

**Published:** 2016-03-28

**Authors:** Samiramis Pourmotabed, Mohammad Jalili

**Affiliations:** ^1^Department of Emergency Medicine, School of Medicine, Hamadan University of Medical Sciences, Hamadan, Iran; ^2^Department of Emergency Medicine, Tehran University of Medical Sciences, Tehran, Iran

## Abstract

Crystal is a synthetic substance with an increasing rate of abuse. It may cause patients to present to the emergency department because of its acute complications. We depict two cases of pneumomediastinum following inhalation of crystal. Both cases had used crystal for recreational purposes. In one case, a young man presenting to the ED with the retrosternal chest pain and neck pain was diagnosed to have pneumomediastinum and pneumopericardium. The other patient presenting with dyspnea and chest pain was shown to have collection of air within mediastinum. Both patients underwent a series of diagnostic evaluations and, after a course of observation, were discharged without a surgical intervention. Patients with chest pain following inhalation of crystal may suffer from this complication.

## 1. Introduction

Crystal methamphetamine (CM) is a colorless, odorless form of d-methamphetamine, a powerful and highly addictive synthetic stimulant [1]. Also known as meth, speed, ice, tina, crystal, tweak, crank, and glass, CM is a methamphetamine powder that can be white, yellow, orange, pink, or brown [2]. It is typically smoked using glass pipes [1]. The abuse of CM has reached epidemic proportions in the United States, with widespread health consequences for a wide segment of the population [2]. Its use is associated with rapid heart rate, increased blood pressure, damage to the small blood vessels in the brain, hyperthermia, convulsions, violent behavior, paranoia, anxiety, confusion, insomnia, and death [1].

Spontaneous pneumopericardium as well as pneumomediastinum, sometimes called pericardial-mediastinal emphysema, is a very rare condition resulting from a multitude of causes: parturition, pulmonary barotrauma, severe cough, asthma, cocaine inhalation, emesis, athletics [3], chlorine gas exposure [4], and certain chemicals inhalation [5, 6].

Isolated cases of pneumomediastinum have been reported following the inhalation of marijuana and cocaine use [7]. Here we describe two cases of spontaneous pneumomediastinum following inhalational consumption of crystal.

## 2. Case Report

### 2.1. Case One

A 24-year-old man presented to the emergency department (ED) complaining of neck and retrosternal chest pain. He reported using inhalational crystal 4 hours ago, when he suddenly felt chest pain and shortness of breath. He did not report any trauma or prolonged cough. He explained that this was the fourth time he was using the drug and he did not have similar problems during previous uses. On physical examination, the vital signs were normal except for slight tachypnea (RR = 18). Oxygen saturation at room air was 94%. There was severe tenderness on neck palpation without crepitation. Chest auscultation was unremarkable.

**Figure 1:**
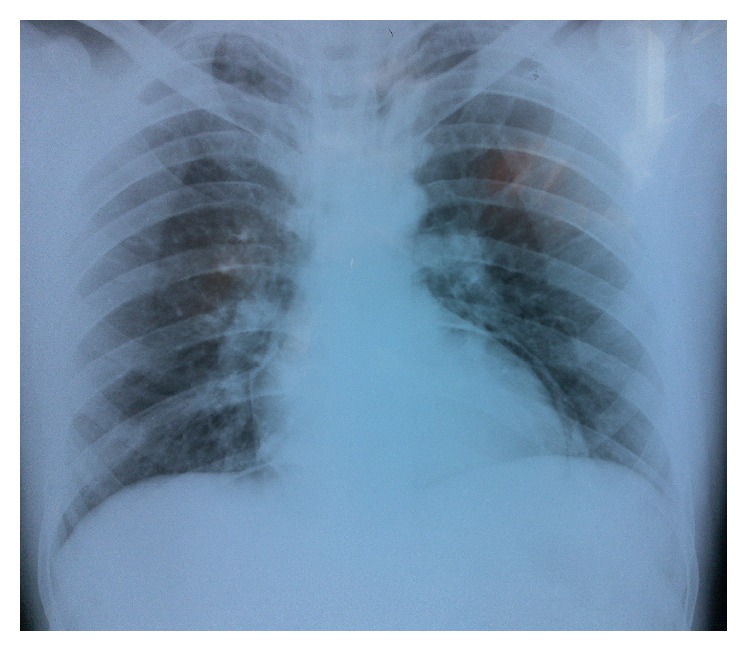


Intravenous line was placed; the patient was put on nasal oxygen while being monitored for heart rate and oxygen saturation. Electrocardiogram (ECG) was performed which was completely normal. The chest X-ray showed accumulation of air within pericardium with no tracheal deviation (Figure 1). Esophageal barium swallow and chest and neck computed tomography (CT) scan were obtained. Barium swallow did not show any contrast leakage. On CT scan, deep neck emphysema was revealed. Chest CT scan also confirmed pneumopericardium and pneumomediastinum. Surgical consultation was requested. The patient was put on observation treatment plan and, after a course of 3 days, was discharged home with no symptoms. In follow-up visit in two days, he was quite symptom-free.

### 2.2. Case Two

A 26-year-old previously healthy man presented with chief complaint of difficulty in breathing and mild retrosternal chest pain. He explained that he had first noticed some dyspnea 3 days ago and that his symptom has gradually worsened. He did not mention cold sweat, pain radiation, fever, cough, or sputum. He mentioned that he had been using crystal for about a year and that he was consuming crystal when his symptoms commenced. On physical examination, he was alert and ill appearing but not in respiratory distress. The vital signs were normal and there was no hypoxemia on pulse oximetry. He looked plethoric. On neck examination, jugular veins appeared normal and there was no tracheal deviation or subcutaneous emphysema. Chest examination findings were negative for decreased or abnormal breath sounds or cardiac murmur.

Oxygenation as well as cardiac monitoring was performed. ECG and arterial blood gas findings were unremarkable. Chest X-ray showed air within the deep neck spaces with no pneumothorax or pneumopericardium. The patient was admitted to the surgery ward and subsequent investigations for pneumomediastinum began. Barium swallow was normal. Chest CT scan showed pneumomediastinum and air in deep neck spaces (Figure 2). The patient underwent observation for 5 days before he was discharged in good health with no residual symptoms.

## 3. Discussion

Synthetic drugs are becoming increasingly popular and are available in various forms. As new drugs are being used, their special impacts on organ systems should be kept in mind when facing those seeking treatment after exposure to them. Most of these drugs are stimulant, as they are sympathomimetic, and may produce some chest syndromes as palpitation or chest pain. Less common complications, however, have been reported and merit consideration such as pulmonary hypertension [8] and cardiomyopathy [9]. One of these possible complications is pneumomediastinum.

This medical emergency can be diagnosed using chest X-ray. However, since obtaining a chest X-ray is not part of the standard of care in these patients, it can be easily missed. In both cases, the disease course was benign and both of them were discharged home after a short observation period. Regarding this benign course, the question remains whether it is acceptable to rely merely on chest X-ray and omit further work-up such as chest CT scan or barium swallow. More information is needed about the exact pathophysiology of this complication in order to decide about suitable diagnostic and therapeutic measures.

## 4. Conclusion

We briefly described two cases of pneumomediastinum following crystal inhalation. Although the natural course of the disease was benign in both cases, we believe that this complication should be kept in mind when facing patients with history of crystal inhalation and chest pain.

## Figures and Tables

**Figure 2:**